# Dynamic changes in thalamic connectivity following stress and its association with future depression severity

**DOI:** 10.1002/brb3.1445

**Published:** 2019-10-25

**Authors:** Xue Zhang, Xuesong Li, David C. Steffens, Hua Guo, Lihong Wang

**Affiliations:** ^1^ Department of Biomedical Engineering Center for Biomedical Imaging Research Tsinghua University School of Medicine Beijing China; ^2^ School of Computer Science and Technology Beijing Institute of Technology Beijing China; ^3^ Department of Psychiatry University of Connecticut School of Medicine Farmington CT USA

**Keywords:** fMRI, mental loading stress, social stress, stress vulnerability, thalamus

## Abstract

**Introduction:**

Tracking stress‐induced brain activity and connectivity dynamically and examining activity/connectivity‐associated recovery ability after stress might be an effective way of detecting stress vulnerability.

**Methods:**

Using two widely used stress paradigms, a speech task (social stress) and a mathematical calculation task (mental loading stress), we examined common changes in regional homogeneity (ReHo) and functional connectivity (FC) before, during, and after the two stressful tasks in thirty‐nine college students. A counting breath relaxation task was employed as a contrast task. ReHo and FC were compared between subjects with higher versus lower depression symptoms (assessed by the Beck Depression Inventory, BDI). We developed a recovery index (RI) based on dynamic changes of ReHo/FC to evaluate individuals' ability to recover from a stressful state. To assess RI's usefulness in predicting future depression severity, BDI was also measured at one‐year follow‐up.

**Results:**

Our results revealed a ReHo decrease after both stressful tasks and a ReHo increase after the relaxation task in bilateral thalamus. The ReHo decrease after both stressful tasks was more significant in the higher BDI than the lower BDI group. Higher ReHo RI of the right thalamus in the higher BDI groups was significantly correlated with lower BDI severity at one‐year follow‐up. Bilateral thalamus also showed increased FC with the default mode network and decreased FC with the executive control network after the stressful tasks.

**Conclusion:**

These findings highlight the importance of tracking resting activity and connectivity of thalamus dynamically for detecting stress vulnerability.

## Graphical abstract

Individuals' recovery ability in the thalamus activity under stress could predict depression severity at one‐year follow‐up.

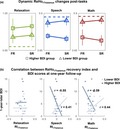

## 1 INTRODUCTION

Stress vulnerability models (Monroe & Simons, 1991) suggest that most individuals, when confronted with daily life stressors, can cope with them efficiently and that they quickly refocus thoughts away from the stress experiences (Robbins, 2005). However, some fail to cope with stress and develop maladaptive responses that can have long‐lasting negative effects on their body and brain (Cohen, Janicki‐Deverts, & Miller, 2007). One's inability to quickly recover from a stressful situation is an important aspect of vulnerability to stress. Dysfunction in stress coping and emotion regulation has been implicated in stress‐related psychiatric disorders (especially depression; Bogdan & Pizzagalli, 2006; Espejo, Hammen, & Brennan, 2012; Hamilton & Gotlib, 2008; Kendler, Karkowski, & Prescott, 1999; Lupien, McEwen, Gunnar, & Heim, 2009). Therefore, it is important to detect individual differences in the ability to recover from stress by examining brain activity/connectivity changes dynamically during and after stress exposures (Stewart, Mazurka, Bond, Wynne‐Edwards, & Harkness, 2013; Wang, Paul, Stanton, Greeson, & Smoski, 2013).

Studies examining stress‐induced neural responses and recovery patterns through post‐stress resting‐state functional magnetic resonance imaging (fMRI) scans are summarized in Table 1. Overall, the intra‐ and inter‐network functional connectivity (FC) of four core networks have been frequently reported altered after acute stress, including the default mode network (DMN), which is related to internally directed self‐referential thought and includes the posterior cingulate cortex (PCC; Clemens et al., 2017; Dimitrov et al., 2018; Quaedflieg et al., 2015; Vaisvaser et al., 2013; Zhang, Hashemi, et al., 2019; Zhang, Huettel, Mullette‐Gillman, Guo, & Wang, 2019); the salience network (SN), which detects bottom‐up salient events and reallocates the attention resources and includes the anterior insula and the dorsal anterior cingulate cortex (Clemens et al., 2017; van Marle, Hermans, Qin, & Fernandez, 2010; Zhang, Hashemi, et al., 2019; Zhang, Huettel, et al., 2019); the executive control network (ECN), which is mainly involved in executive function and includes the dorsolateral prefrontal cortex (dlPFC) and intraparietal lobe (IPL; Vaisvaser et al., 2013; Zhang, Huettel, et al., 2019); and the limbic system, which processes emotion information and directs it to the frontal cortex for decision‐making and includes the amygdala and the thalamus (Dimitrov et al., 2018; van Marle et al., 2010; Maron‐Katz, Vaisvaser, Lin, Hendler, & Shamir, 2016; Quaedflieg et al., 2015; Vaisvaser et al., 2013; Zhang, Huettel, et al., 2019). However, the main findings of these studies are quite divergent. Discrepancies among these studies could be due to different stressful tasks used across studies and individual differences in response to different types of stressors. Different seed regions selected for FC analysis may also contribute to the inconsistent findings in the literature. The seed‐based FC analysis relies on a priori hypotheses and neglects potential changes unrelated to the seeds. It would be beneficial to compare changes in voxel‐wise activity over time (such as regional homogeneity‐ReHo and amplitude of low‐frequency fluctuations‐ALFF) to fully uncover stress‐induced changes in the brain. ReHo and ALFF are two approaches that reveal regional functional activity, and both are reliably correlated with cerebral blood flow in most of the brain cortex (Li, Zhu, Childress, Detre, & Wang, 2012). ReHo is more sensitive to depict regional dysfunction because it accounts for the dynamic features of the time courses of neighboring voxels (An et al., 2013).

**Table 1:** An overview of resting‐state studies examining the stress response and recovery in the brain

References	Task	Increased FC	Decreased FC
van Marle et al. (2010)	Violent movie clips	Amygdala‐dACC Amygdala‐anterior insula Amygdala‐LC	\
Quaedflieg et al. (2015)	Mental arithmetic task	Amygdala‐right para‐hippocampal gyrus	Amygdala‐left vlPFC Amygdala‐bilateral vPCC Amygdala‐culmen Amygdala‐bilateral cuneus
Vaisvaser et al. (2013)	Mental arithmetic task	PCC‐mPFC PCC‐thalamus PCC‐caudate PCC‐IPL Hippocampus‐left amygdala Hippocampus‐MTG	PCC‐posterior insula PCC‐lingual gyrus
Maron‐Katz et al. (2016)	Mental arithmetic task	Thalamus‐frontal lobe Thalamus‐bilateral temporal lobe Thalamus‐right parietal lobe	Cross‐hemispheral parietal‐temporal lobe
Clemens et al. (2017)	Cyberball task	DMN‐SN DMN‐sensorimotor regions DMN‐higher‐order visual areas	\
Dimitrov et al. (2018)	Modified montreal imaging stress task	Male nonresponders Bilateral amygdala‐PCC/precuneus	\
Zhang, Huettel, et al. (2019)	Mental arithmetic task	Regional connectivity of Amygdala Anterior insula rdlPFC	Regional connectivity of PCC
Zhang, Hashemi, et al. (2019)	Cold pressor task and mental arithmetic task	Intra‐network connectivity of SN	Intra‐network and global connectivity of DMN

Abbreviations: dACC, dorsal anterior cingulate cortex; DMN, default mode network; LC, locus coeruleus; IPL, inferior parietal lobule; mPFC, medial prefrontal cortex; MTG, middle temporal gyrus; PCC, posterior cingulate cortex; rdlPFC, right dorsal lateral prefrontal cortex; SN, salience network; vlPFC, ventral lateral prefrontal cortex; vPCC, ventral posterior cingulate cortex.

To our knowledge, there are no published studies that have examined core/shared neural correlates of stress using different types of stressors and tracked changes in whole‐brain voxel‐wise resting‐state activity over time (i.e., before, during, and after the stressful tasks). In addition, despite evidence of the link between depression etiology and stress recovery ability (higher depressive symptoms are associated with poorer/slower cortisol and heart rate recovery post‐stress; Shapero, McClung, Bangasser, Abramson, & Alloy, 2017), few studies have focused on dynamic brain connectivity or activity patterns related to recovery from stress. No quantitative measures to evaluate recovery ability have been proposed or examined in terms of its association with the risk of subclinical depression. Therefore, multiple stressors, dynamic whole‐brain voxel‐wise neural activity changes, and the association of depression and stress are the key areas of focus of the present study.

Among the cortical and subcortical brain regions involved in stress response and stress recovery, the thalamus is unique, as it is a coordination center that transfers information between subcortical regions and the cortices. The thalamus is also involved in memory (Johnson & Ojemann, 2000) and sleep regulation (Min, 2010), probably through sensory arousal effect, which can be influenced by stress and depression. Several studies have linked thalamus with depression. A postmortem study found elevated neuron number in the thalamus in depression (Young, Holcomb, Yazdani, Hicks, & German, 2004). An electrical stimulation study (Fisher et al., 2010) reported an increased likelihood of depressive symptoms by stimulating the anterior nucleus group of the thalamus (suppressing thalamus activity). Notably, the dysfunction of limbic‐thalamo‐cortical circuit involving the amygdala, the mediodorsal (MD) nucleus of the thalamus, and the medial prefrontal cortex (mPFC) has been implicated in depression etiology (Drevets et al., 1992; Holmes et al., 2012) and stress response (Park et al., 2018). As the thalamus is a vital relay center between the amygdala and the mPFC and gateway for information filtering, the activity/connectivity of the thalamus may reflect one's attention focus under stress (increased stress‐related sensory information transfer) and recovery of the homeostasis (decreased stress‐related information delivery). Therefore, the measure of dynamic changes of thalamus activity/connectivity overtime after stress might be an important imaging marker of stress adaptation outcome.

The current study had two main goals: to explore the shared neuropathways in response to different stressors to confirm the core neural systems related to acute stress; and to develop a quantitative measure to evaluate individuals' ability to recover from stress and to test the hypothesis that the recovery ability could predict future depression severity. To this end, we conducted three fMRI sessions with two stressful tasks (speech task and math calculation task to induce social stress and mental loading stress respectively) and one relaxation task (breath counting). We examined regional (regional homogeneity, ReHo) and distant FC changes in a pret‐ask resting‐state run, during task runs, and in a post‐task resting‐state run to evaluate how our brain responded and recovered intrinsically from a stressful state to the homeostasis to analog daily life situations. Common changes in resting‐state activity/connectivity induced by both stressful tasks were examined. We further examined differences in the stress‐related activity/connectivity between individuals with higher depressive symptoms and those with lower depression symptoms assessed by the Beck Depression Inventory (BDI). To assess individuals' recovery ability from a stressful state to pre‐stress baseline, a recovery index (RI) was developed based on the dynamic ReHo and FC changes post‐stress, and the relationship of RI with depression severity at one‐year follow‐up was also examined. We hypothesized that common changes would be observed under the two stressful tasks in regions of the DMN, SN, ECN, or the limbic system, and the dynamic neural activity changes in the thalamus induced by stressful tasks would be significantly greater in the higher BDI group than the lower BDI group. We also expected the RI to be associated with future depression severity.

## 2 METHODS

### 2.1 Subjects

Forty‐five young college students (mean age = 22.9 years, *SD* = 2.67 years, 23 males) were recruited through advertisement during a class break. To ensure that subclinical depression subjects would be included in the study, our advertisements targeted those who had a self‐concern about depression and who felt themselves vulnerable to stress. Subjects who had any history of head injury, psychiatric or neurological disorders, or other major medical conditions were excluded. This study was approved by the local Medical Ethics Committee at Tsinghua University School of Medicine, and written informed consent was obtained from all subjects. Each subject was asked to participate in three consecutive MRI scans (two stressful tasks and one relaxation task) with 5–10 days between any two sessions. The two stressful tasks and one relaxing task were assigned to subjects in a counterbalanced order. Subjects were scanned at about the same time of a day across MRI sessions to avoid time fluctuation effects on cortisol level.

### 2.2 Psychological assessments

The 13‐item BDI (Beck & Beck, 1972) was used to assess depression symptoms, and the questionnaire was completed during the class break before the MRI scan for all subjects. Each subject also completed the BDI one year later the completion of MRI scans. For the 13‐item short‐form BDI, 0–4 corresponds to none or minimal depression, 5–7 to mild depression, 8–15 to moderate depression, and 16 or higher to severe depression (Fiske, Kasl‐Godley, & Gatz, 1998). Although these subjects were not clinically diagnosed as major depression, 16 subjects had BDI score over 8, suggesting we might have included subclinical subjects (Cuijpers & Smit, 2004) in our study sample, which fits well with our study purpose. In order to identify subclinical depression‐related brain activity changes, subjects were subdivided into lower BDI group (0 ≤ BDI ≤7, *n* = 29, none to mild depression) and higher BDI group (8 ≤ BDI ≤ 17, *n* = 16, moderate to severe depression). The highest BDI score among all subjects was 17. We chose 7 (the boundary for mild and moderate depression) as the cutoff for group definitions to ensure similar BDI ranges in the two groups. To be noted, we only included subjects with negligible motion during scanning for all analyses (see Section 2.5.2 for the exclusion criteria), that resulted in 25 subjects in the lower BDI group and 14 subjects in the higher BDI group. State anxiety was assessed using the Spielberger State Anxiety Inventory (STAI state; Spielberger, Gorsuch, Lushene, Vagg, & Jacobs, 1983) pre‐ and post‐tasks.

### 2.3 Experiment design

**Figure 1:**
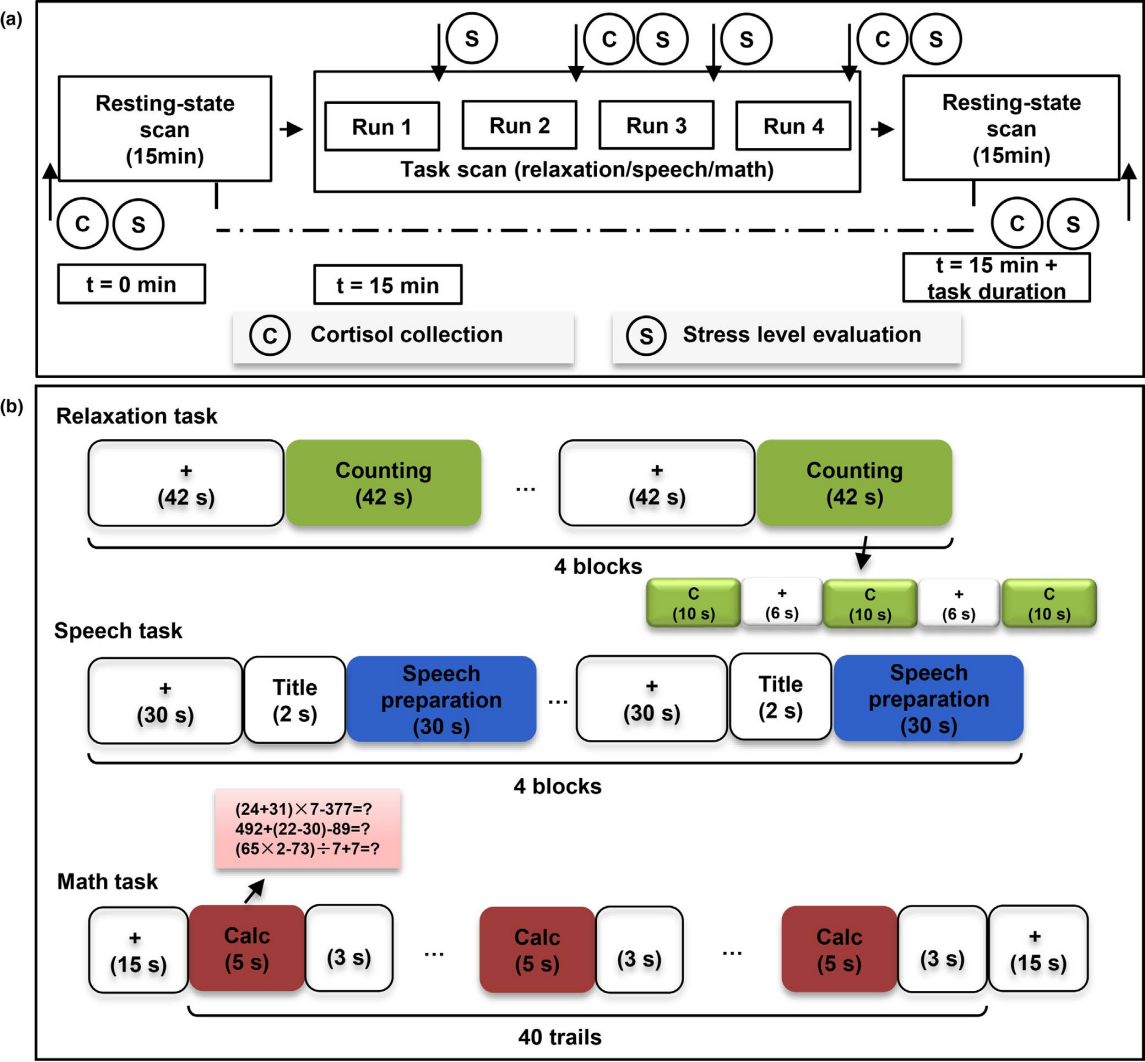
(a) The scanning procedures for two stressful tasks and one relaxation task, dotted line marks the focus of this study: comparison of resting‐state activity/connectivity between post‐ versus pre‐tasks. (b) Task designs for two stressful tasks and one relaxation task. C: counting

Experimental procedures and task designs are shown in Figure 1a,b. Two widely used stress paradigms were selected: a speech task to induce social stress and a math calculation task to cause mental loading stress (Figure 1b). Counting from 1 to 10 along with breathing was employed as a relaxing task (Figure 1b). Each task consisted of 4 task runs; one 15‐min resting‐state run was acquired pre‐ and post‐task runs (Figure 1a). Task details could be found in the Supporting Information.

In order to confirm that the stressful and the relaxing tasks could induce stressful and relaxation states successfully, subjective ratings on stress level and cortisol level were collected (Figure 1a). The stress level was evaluated at baseline (pre‐task), during the task (immediately after each run), and after the task (post‐task) using a visual analog scale by an integer between 1 and 10, with 10 corresponding to the most catatonic state they had experienced before. Saliva samples were collected four times: at baseline (pre‐task), during the task (after the second and fourth task run), and after the task (post‐task) by instructing subjects to chew a synthetic swab (Sarstedt) for 1 min. The saliva samples were stored at −20°C until they were assayed by Kangjiahongyuan Biotechnology Co., Ltd.

### 2.4 Data acquisition

MRI data were collected using a 3.0‐Tesla Philips Achieva scanner located at the Center for Biomedical Imaging Research at Tsinghua University. High‐resolution 3D T1‐weighted anatomical images were collected transversely using a MPRAGE sequence with TR = 7.5 ms, TE = 3.7 ms, FOV = 23.0 cm^2^, matrix = 230 × 230, 180 slices, and voxel size = 1 mm^3^. Functional images were acquired axially using an echo‐planar imaging sequence with TR = 2,000 ms, TE = 30 ms, FOV = 22.4 cm^2^, matrix = 64 × 64, 34 slices, and voxel size = 3.5 × 3.5 × 3.5 mm^3^ with 0.5 mm gap between slices. During the 15‐min resting‐state fMRI scans, subjects were asked to fixate their eyes on the “+” at the center of the screen without thinking anything systematically. Task‐related fMRI data were acquired with 168 trials for the relaxing task run, 124 trials for the speech task run, and 175 trials for the math task run. Physiological (cardiac and respiratory) data were continuously collected throughout each fMRI session using the scanner's built‐in monitoring system.

### 2.5 Data analysis

#### 2.5.1 Behavioral and physiological measures

Demographic and psychometric characteristics by BDI group are summarized in Table 2. Self‐evaluated stress level, heart rate, and respiratory rate were analyzed through a 3 tasks (speech, math, and relaxation) × 6 time‐points (pre‐task resting‐state run, 4 task runs, and post‐task resting‐state run) two‐way repeated‐measures ANOVA. The 3 tasks × 4 time‐points (pre‐task, during the second and fourth tasks, and end of the scan) for cortisol measures and 3 tasks × 2 time‐points (pre‐ and post‐task) for STAI state two‐way repeated‐measures ANOVA were also computed. A paired *t* test was applied to assess the post‐ versus pre‐task change in stress level, heart rate, respiratory rate, and STAI state as post hoc tests. Cortisol levels of the second, third, and fourth measures were averaged to compare with the baseline measurement using a paired *t* test.

**Table 2:** Subjects' demographics and depression severity scores

	*N*	Age	Gender (F/M)	Current BDI	BDI 1‐year‐later
All	39	23.10 ± 2.70	20/19	6.08 ± 4.73	4.11 ± 3.63
Higher BDI	14	22.80 ± 2.42	9/5	11.36 ± 3.13	5.52 ± 4.84
Lower BDI	25	23.20 ± 2.89	11/14	3.12 ± 2.20	3.31 ± 2.52
*p*		.65		<.001	.07

Note

*p*‐value indicates the difference between the higher and the lower BDI group.

Abbreviation: BDI, Beck Depression Inventory.

The current and one‐year‐later BDI scores were analyzed through a 2 groups (higher BDI, lower BDI) × 2 time‐points (current, one‐year follow‐up) two‐way mixed ANOVA. Pearson's correlation coefficient between the current and future BDI scores was also calculated.

#### 2.5.2 FMRI data preprocessing

Standard functional data preprocessing procedures including slice timing, realignment, and motion parameters regression were carried out using the default parameters of DPABI V1.3 (http://rfmri.org/DPABI) for resting‐state and task fMRI data. Subjects with head motion exceeding 3.0‐mm translation or 3.0‐degree rotation were excluded from further analysis. Data from six subjects were excluded using these criteria. Additional denoising steps were taken for resting‐state data, physiological noise was corrected with both RETROICOR (Glover, Li, & Ress, 2000) and RVHRCOR (Chang & Glover, 2009), five principal components derived from the time series of white matter and cerebrospinal fluid (CSF) were regressed out, and the data were bandpass filtered between 0.01 and 0.1 Hz. After preprocessing, the functional images were normalized to the standard MNI space using DARTEL (Ashburner, 2007) and resampled into 3‐mm isotropic voxels (DPABI default). Finally, all fMRI images were spatially smoothed by a 3D‐Gaussian kernel with 4 mm full width at half maximum.

#### 2.5.3 Regional homogeneity evaluation

To assess changes in regional neural activity synchronization under stressful and relaxing conditions, we calculated pre‐, during‐ (4 task runs), and post‐task whole‐brain voxel‐wise regional homogeneity (ReHo) for each subject. Kendall's correlation coefficient (KCC) of the time series of a given voxel with its nearest neighboring voxels (26 voxels) was used to estimate the extent of regional synchronization for ReHo (Zang, Jiang, Lu, He, & Tian, 2004). Voxel‐wise ReHo was divided by the global mean ReHo.

To identify common ReHo changes under the two stressful tasks, a conjunction analysis was conducted between the two stressful tasks using the pre‐stress relative to post‐stress resting‐state contrast. Significant regions from the conjunction analysis were extracted as regions of interest (ROIs), and ReHo of the ROIs under three tasks was entered into a two‐way repeated‐measures ANOVA (3 tasks × 6 time‐points. 3 tasks: speech, math, and relaxation; 6 time‐points: pre‐task resting‐state run, 4 task runs, and post‐task resting‐state run) to examine the manipulation effects of the stressful and relaxing tasks. Only ROIs showed ReHo changes in the opposite direction under stressful and relaxing tasks were considered to be related to ones' stressful state and were retained for further analyses.

Considering that the altered resting‐state activities might be the remaining impact (after effect) of each task, the total ReHo change [during task (4 task runs) + post‐task resting run] relative to the pre‐task resting run was calculated and compared between the higher BDI and lower BDI groups (two‐sample *t* test), and its relationship with BDI scores at each group was also examined respectively using Pearson's correlation analysis.

#### 2.5.4 Functional connectivity assessments

Functional connectivities of the selected ROIs to the whole brain were calculated and transformed by Fisher's Z normalization using DPABI as well. Conjunction analysis was also conducted to identify shared post‐ versus pre‐stress resting‐state FC alterations, to show a full picture of the brain's network interactions induced by stress.

To be noted, the significant level for all whole‐brain voxel‐based statistical analyses was set at *p* < .05 with FDR correction (Benjamini & Hochberg, 1995) for multiple comparisons.

#### 2.5.5 Recovery index

To assess subjects' ability to recover from stress, each 15‐min resting scan was divided into two 7.5‐min segments (first half run: FR; second half run: SR) for pre‐ and post‐task resting‐state runs, ReHo for the two segments was calculated, respectively. A 2 groups (higher BDI, lower BDI) × 2 time frames (FR, SR) × 3 tasks (speech, math, and relaxation) three‐way mixed ANOVA on post‐task ReHo was conducted to show the distinct dynamic trajectories of the lower BDI and the higher BDI individuals.

A recovery index (RI) was defined as the ReHo difference between SR and FR in post‐task resting‐state scan relative to pre‐task resting‐state scan (Equations 1 and 2). We assumed that the pre‐task ReHo was relatively temporally stable. We aimed to define RI with a higher value indicating a better recovery ability, and the equation was modified for each condition: increased post‐task ReHo and decreased post‐task ReHo. If the ReHo increased after stress exposure, a strong recovery ability would be implicated by a decreased ReHo in the SR in the post‐stress resting run, RI was defined as the numeric difference between the FR and SR (Equation 1), while if the ReHo decreased after stress exposure, a strong recovery ability would be implicated by an increased ReHo in the SR in the post‐stress resting run, and RI was defined as the numeric difference between the SR and FR (Equation 2). Recovery index of FC was also calculated by replacing ReHo in the Equations 1 and 2 and analyzed through the three‐way ANOVA as described above for the ReHo recovery index. We explored the relationship between the RI and future BDI using Pearson's correlation analysis.

(1) $${\text{RI}}_{\text{reho increase}}=R_{2}\left({\text{ReHo}}_{\text{FR}}-{\text{ReHo}}_{\text{SR}}\right)-R_{1}\left({\text{ReHo}}_{\text{FR}}-{\text{ReHo}}_{\text{SR}}\right)$$

(2) $${\text{RI}}_{\text{reho decrease}}=R_{2}\left({\text{ReHo}}_{\text{SR}}-{\text{ReHo}}_{\text{FR}}\right)-R_{1}\left({\text{ReHo}}_{\text{SR}}-{\text{ReHo}}_{\text{FR}}\right)$$

*R*_1_: pre‐task resting run; *R*_2_: post‐task resting run.

For all group‐level analyses, age, gender, and educational years were entered as nuisance regressors.

## 3 RESULTS

### 3.1 Behavioral and physiological measures

**Figure 2:**
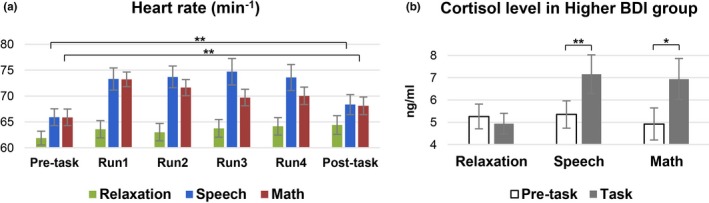
(a) Stress‐induced heart rate changes over time from the pre‐task resting run, 4 task runs to the post‐task resting run under the two stressful tasks and the relaxation task. (b) Cortisol responses (mean change across‐task and post‐task vs. pre‐task baseline) under the two stressful tasks and the relaxation task in the higher Beck Depression Inventory group. *p < .05, **p < .01

All statistical results from the ANOVA analyses are shown in the Supporting Information and Table S1. Here, we only report the shared post‐ versus pre‐task changes under the speech and the math tasks. There was a significant interaction of task × time in the heart rate (*F*_(10, 380)_ = 13.56, *p* < .001). Post hoc tests revealed that heart rate increased after both speech (*T*_(38)_ = 3.65, *p* = .0004) and math tasks (*T*_(38)_ = 3.76, *p* = .0003), but not after the relaxing task (*T*_(38)_ = 1.29, *p* = .10; Figure 2a). A significant interaction effect of time × task on cortisol was found in the higher BDI group (*F*_(6, 78)_ = 2.28, *p* = .04), but not in the lower BDI group. The cortisol levels in the higher BDI group (Figure 2b) increased during both stressful tasks (*T*_(13)_ = 3.25, speech: *p* = .003; *T*_(13)_ = 2.06, math: *p* = .03). These results validate the effect of stressful and relaxing tasks and demonstrate the differences in behavioral and physiological responses to stress between the higher and lower BDI groups.

There was a significant interaction effect of time × BDI group (*F*_(1, 74)_ = 11.71, *p* = .001) in the 1‐year‐later BDI change. Specifically, individuals in the higher BDI group showed decreased BDI after 1 year (paired *t* test, *T*_(13)_ = 2.12, *p* = .03, Figure S2b) than those in the lower BDI group. BDI score at one‐year follow‐up was correlated with the current BDI score among all subjects (*r*_(37)_ = 0.38, *p* = .02; Figure S2a), in the lower BDI group (*r*_(23)_ = 0.47, *p* = .02), but not in the higher BDI group (*r*_(12)_ = 0.25, *p* = .38), indicating that higher BDI could be a temporary state and it does not necessarily predict the future depression severity.

### 3.2 Common changes in ReHo after the two stressful tasks and the relationships with depression severity

**Figure 3:**
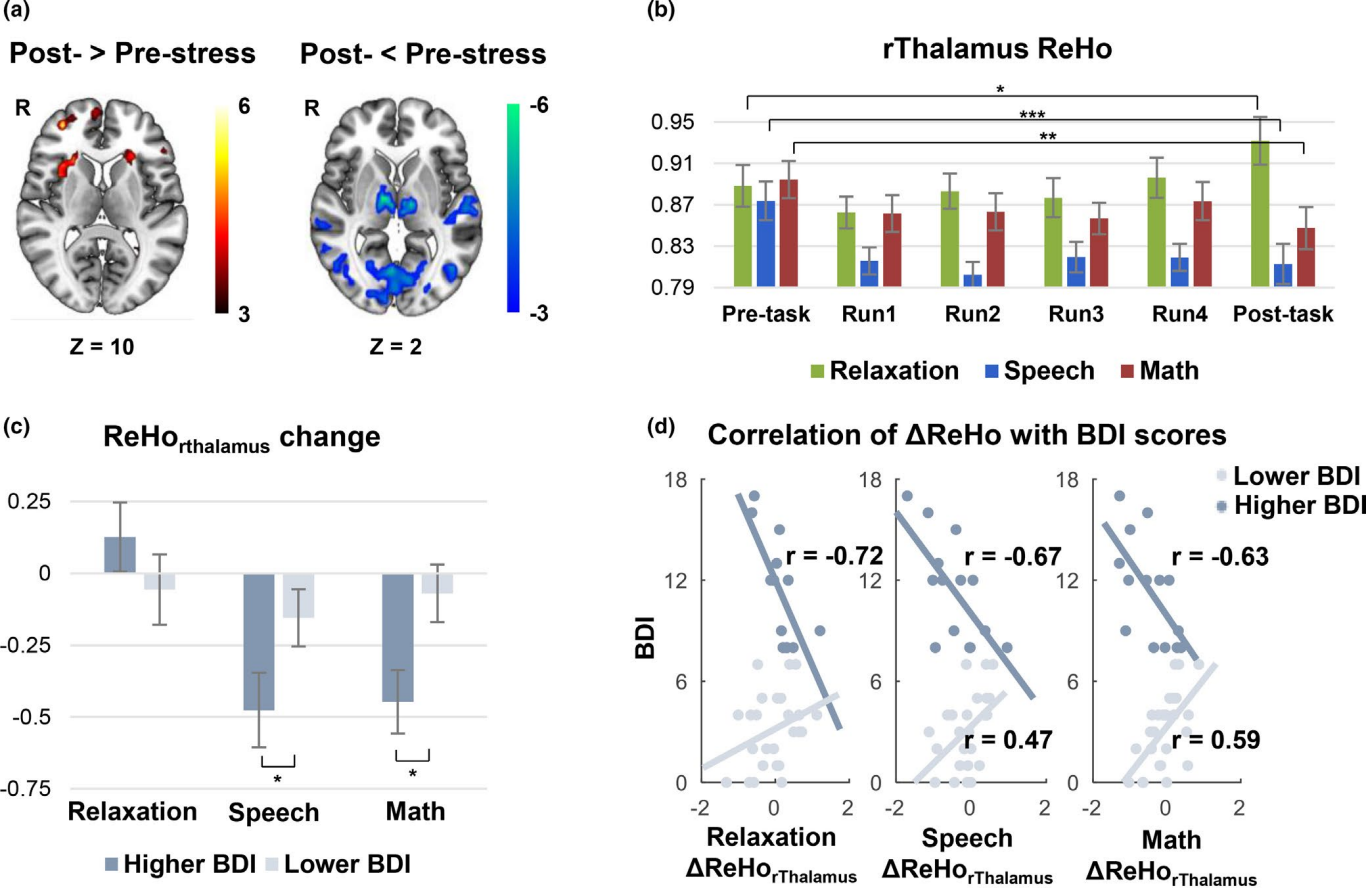
(a) Shared changes in regional homogeneity (ReHo) under the speech and math tasks from conjunction analysis. The right anterior insula and right dorsolateral prefrontal cortex showed increased ReHo, while bilateral thalamus, bilateral middle temporal gyrus, and visual cortex showed decreased ReHo under both stressful tasks. (b) Stress‐induced ReHo changes in the right thalamus (rThalamus) from the pre‐task resting run, 4 task runs, and post‐task resting run under the two stressful tasks and the relaxation task. Decreased ReHo was observed during and after the two stressful tasks while increased ReHo was found after the relaxation task. (c) ReHo changes in the rThalamus among the higher and lower Beck Depression Inventory (BDI) groups across three tasks. The ReHo decrease in the rThalamus during and after stressful tasks was significant greater in the higher BDI group than that in the lower BDI group. (d) The ReHo change in the rThalamus was positively correlated with the current BDI scores in the lower BDI group but negatively correlated with the current BDI scores in the higher BDI group in both speech and math tasks. BDI scores were negatively correlated with rThamulus ReHo change even after the relaxation task in the higher BDI group. *p < .05, **p < .01, ***p < .001

Conjunction analysis in ReHo changes pre‐ versus post‐speech and math conditions showed increased ReHo in the right anterior insula and the right dlPFC, and decreased ReHo in the bilateral thalamus, the bilateral middle temporal gyrus, and the visual cortex (Figure 3a). Pre‐ and post‐relaxation resting‐state ReHo of the above regions were extracted and compared. We detected opposing findings only in the bilateral thalamus, with decreased ReHo after the stressful tasks, and increased ReHo after the relaxation task (*T*_(38)_ = 1.85, *p* = .04). Considering the left and right thalamus showed similar results, we hereafter present the results for the right thalamus in the manuscript and include the results for the left thalamus in Figures S3 and S4. ANOVA analysis revealed a significant interaction of time × task in the ReHo of the right thalamus (*F*_(2, 76)_ = 10.98, *p* < .001). Figure 3b shows ReHo changes in the right thalamus at the pre‐task resting‐state run, across 4 task runs, and at the post‐task resting‐state run. Decreased ReHo was observed during both stressful tasks, suggesting that reduced ReHo in the post‐stress resting run (speech: *T*_(38)_ = 3.85, *p* = .0002; math: *T*_(38)_ = 2.67, *p* = .006) might be a continuous effect of the stressful tasks.

ReHo decrement, which was calculated as the total ReHo change during‐ and post‐task relative to pre‐task, was found significantly greater in the higher BDI group than that in the lower BDI group (speech task *T*_(37)_ = 1.78, *p* = .04, math task *T*_(37)_ = 1.91, *p* = .03; Figure 3c). Interestingly, we found the greater reduction of ReHo in the right thalamus was significantly correlated with higher BDI scores in the higher BDI group (speech: *r*_(12)_ = −0.67, *p* = .01; math: *r*_(12)_ = −0.63, *p* = .02), but was correlated with lower BDI scores in the lower BDI group (speech: *r*_(23)_ = 0.47, *p* = .02; math: *r*_(23)_ = 0.59, *p* = .002; Figure 3d).

### 3.3 Common changes in the right thalamus connectivity post‐stress

**Figure 4:**
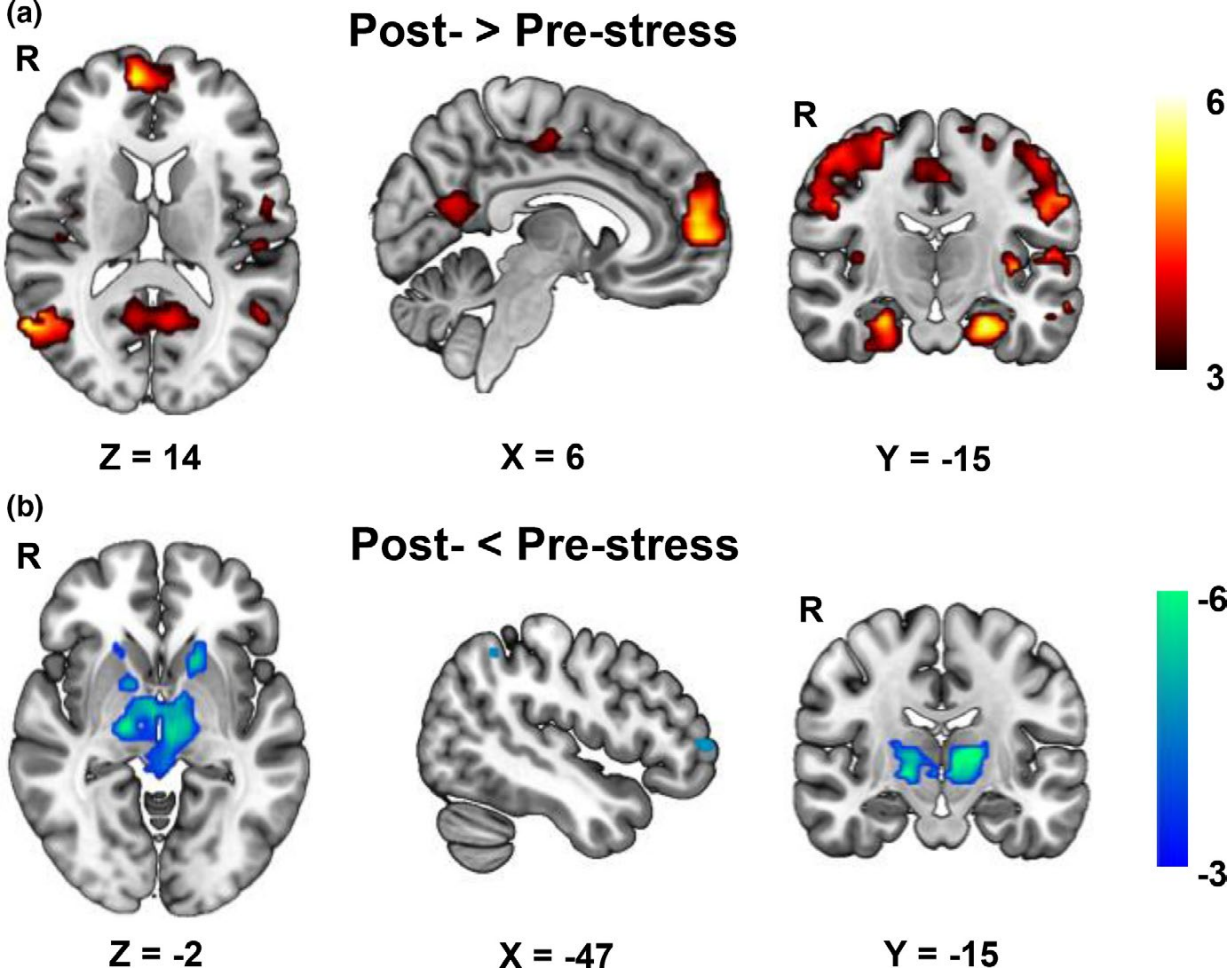
Common functional connectivity changes of the right thalamus (rThalamus) induced by the speech and math tasks from conjunction analysis. (a) Increased functional connectivity of the rThalamus with medial prefrontal cortex (mPFC), middle cingulate cortex (mid‐CC), posterior cingulate cortex (PCC), bilateral superior temporal gyrus, bilateral hippocampal and para‐hippocampal gyrus, sensory motor areas, and (b) decreased functional connectivity of the rThalamus with the left dorsolateral prefrontal cortex (ldlPFC), left intraparietal lobe (lIPL), putamen, and thalamus itself

To further understand the role of ReHo reduction of the right thalamus in stress vulnerability, we examined the common FC changes of the right thalamus seed with voxels of the whole brain after the two stressful tasks. We found increased FC of the right thalamus with mPFC, middle cingulate cortex (mid‐CC), PCC, bilateral superior temporal gyrus, bilateral hippocampal and para‐hippocampal gyrus, and sensory motor areas (Figure 4a), and decreased FC with left dlPFC, left intraparietal lobe (IPL), and thalamus itself (Figure 4b).

### 3.4 Recovery ability of the right thalamus activity post‐stress

**Figure 5:**
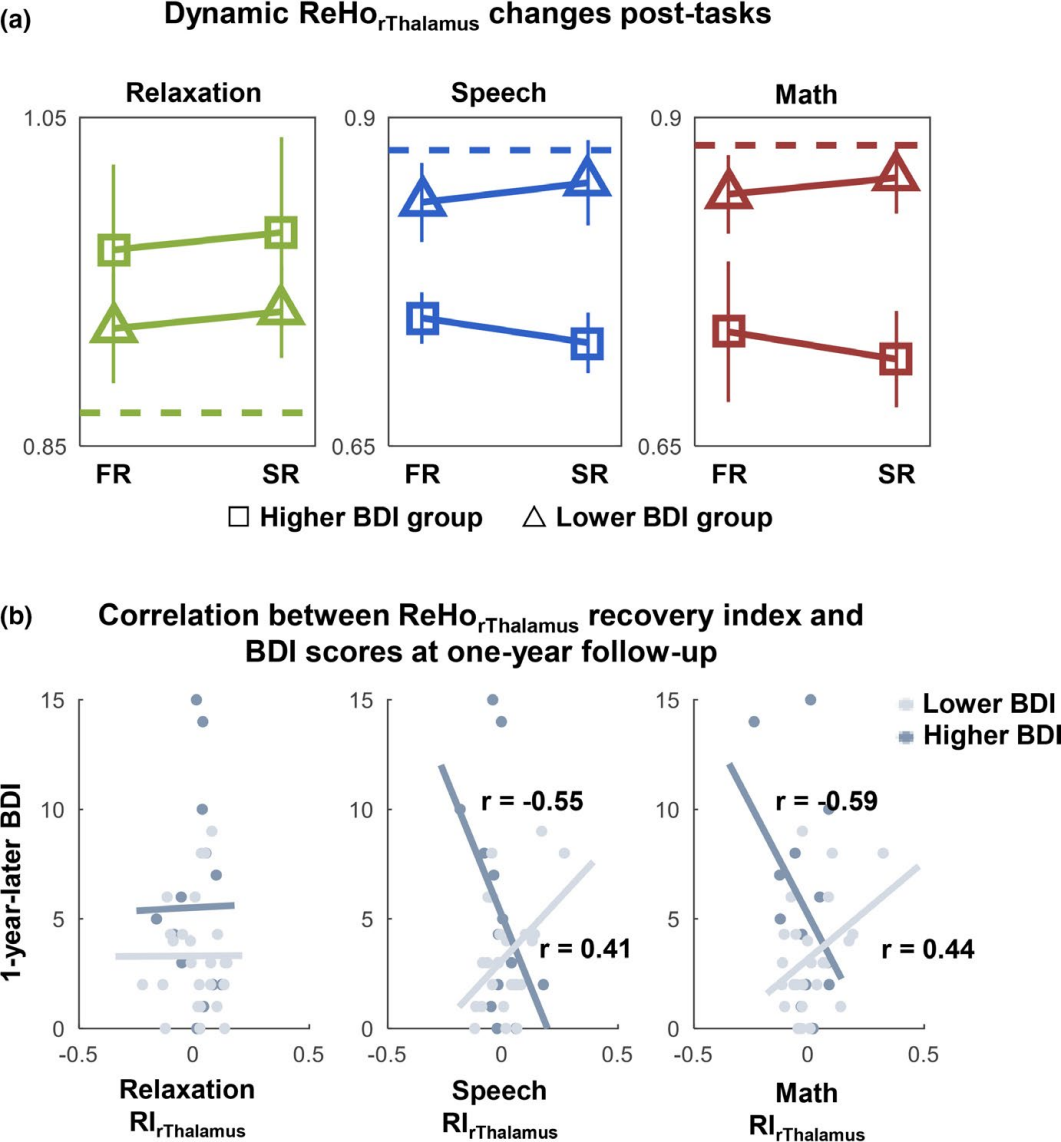
(a) Dynamic post‐task (relaxation, speech, and math tasks) regional homogeneity (ReHo) changes in the right thalamus (rThalamus) in the higher and lower Beck Depression Inventory (BDI) groups. FR = the first half resting run and SR = the second half resting run after each task, dotted lines indicate the pre‐task ReHo in the rThalamus. (b) The ReHo recovery index (RI) in the rThalamus after the two stressful tasks was positively correlated with BDI scores at one‐year follow‐up in the lower BDI group but negatively correlated with the one‐year‐later BDI scores in the higher BDI group

The two BDI groups (higher and lower BDI) × 3 tasks (speech, math, relaxation) × 2 post‐task time segments (FR, SR) three‐way ANOVA showed a significant interaction effect of task × BDI group (*F*_(2, 222)_ = 5.34, *p* = .005) in the right thalamus ReHo. However, there was no significant main or interaction effect of time segments. Paired *t* test did not show any significant ReHo changes between the FR and SR in either higher BDI group or lower BDI group although the higher BDI group tended to decrease more while the lower BDI group tended to increase to the pre‐stress baseline (Figure 5a). However, ReHo RI of the right thalamus was correlated with BDI scores at one‐year follow‐up (Figure 5b) although not with current BDI scores. Furthermore, the higher and lower BDI groups showed opposite directions in RI correlation with future BDI. For the lower BDI group, higher RI was correlated with higher BDI (speech: *r*_(12)_ = 0.41, *p* = .04; math: *r*_(12)_ = 0.44, *p* = .03), while for the higher BDI group, higher RI was correlated with lower BDI at one‐year follow‐up (speech: *r*_(23)_ = −0.55, *p* = .04; math: *r*_(23)_ = −0.59, *p* = .03), and no correlation was found for the relaxation task. The connectivity RIs of the thalamus were not significantly different between the higher and lower BDI groups and were not correlated with BDI scores at one‐year follow‐up either.

Neither ReHo nor FC of the right or left thalamus was significantly correlated with the anxiety level measured by STAI state before or after the stressful tasks.

## 4 DISCUSSION AND CONCLUSION

In this experiment, two stressful tasks and one relaxation task were implemented to examine brain activity/connectivity changes under different mental states and its associations with depression vulnerability. We focused on identifying common changes induced by the two stressful tasks and examined whether higher versus lower depression symptom severity (measured by BDI) groups differed in the recovery of stress‐induced changes over time. We found many regions showed commonly increased and decreased ReHo after stressful tasks, yet only the bilateral thalamus showed increased ReHo after the relaxation task, and the change was opposite to the findings after the stressful tasks. Furthermore, the ReHo decrease after stressful tasks was significantly greater in the higher BDI group than that in the lower BDI group. We further found that in the higher BDI group, lower recovery ability (recovery index) on resting‐state ReHo of the right thalamus was associated with higher depression severity at one‐year follow‐up. Moreover, significantly higher FC between bilateral thalamus and the DMN (including hippocampus) and lower FC between bilateral thalamus and the ECN was observed post‐stress. These results highlight the important role of the thalamus in detecting stress and depression vulnerability that has been relatively neglected in previous studies.

ReHo in the right anterior insula (a key node of SN) and right dlPFC (a key node of ECN) increased after both stressful tasks, which is consistent with our previous findings (Zhang, Huettel, et al., 2019) and others (Alvarez et al., 2015; Dedovic, D'Aguiar, & Pruessner, 2009; Sinha, Lacadie, Constable, & Seo, 2016; van der Werff, Berg, Pannekoek, Elzinga, & Wee, 2013). We suggest that when facing stress, emotional salience increases along with increased activity of the right anterior insula, which subsequently alerts prefrontal cortex for emotion regulation. Therefore, an increase in dlPFC activity is necessary to enhance cognitive processing to adapt to the stressful state, similar to the increase in cortisol stress hormones (Yuen et al., 2009).

Findings in the bilateral thalamus revealed reduced ReHo after the stressful tasks but elevated ReHo after the relaxing task. The opposite changes in the thalamus under stress and relaxation highly support the hypothesis that thalamus activity could sensitively reflect the outcome of stress coping. Decreased ReHo in the thalamus during and after stressful tasks suggests a prolonged effect of stressors on the brain. Individuals with higher BDI tended to show a greater decrease in ReHo of thalamus than those with lower BDI, and interestingly, greater ReHo reduction was correlated with lower BDI scores in the lower BDI group, while it was associated with higher BDI scores in the higher BDI group. For lower BDI individuals, the highest BDI score was 7, which is not meaningful clinically. For the higher BDI group, individuals with higher BDI might represent prodromal/subclinical depression, and excessively reduced ReHo of thalamus could be an outcome of their reduced stress coping ability. Therefore, reduced ReHo in the thalamus could reflect stress vulnerability, which is further evidenced by the finding that in the higher BDI group, poor recovery of the reduced ReHo in the thalamus was associated with higher BDI score at one‐year follow‐up.

Regarding the post‐stress recovery ability in the right thalamus ReHo, we found no difference in the recovery index between the higher and lower BDI groups. Considering the BDI in the higher BDI group decreased significantly one year later, it is understandable that no recovery ability difference was detected at baseline. The recovery index was associated with BDI scores at the one‐year follow‐up, which indicates that the recovery index is not an index of the current depressive state; instead, it is an indicator or risk factor for future depression severity, as we hypothesized. Notably, the recovery index was negatively correlated with the 1‐year‐later BDI score in the higher BDI group as expected, indicating that a poorer recovery ability predicts future greater depression severity. The recovery index was positively correlated with the 1‐year‐later BDI scores in the lower BDI group. We speculate that a slower recovery of reduced thalamus ReHo in the lower BDI group might be due to stress‐related memory consolidation to prepare the brain and the body for future similar stressful events (Dimitrov et al., 2018; Roozendaal, McEwen, & Chattarji, 2009) given the connectivity between the thalamus and bilateral hippocampal and para‐hippocampal gyrus increased after stressful tasks and the fact that stress‐related hormones (norepinephrine and cortisol) directly affect the activity of hippocampal gyrus (Roozendaal et al., 2009). Together with the finding of the opposite correlation between ReHo decrease and depression severity, the opposite relationship observed for RI between the two groups might accentuate the dynamic transition of thalamus's function in the development of psychiatric disorders as greater activity changes facilitate the stress adaptions in normal condition but indicate the abnormal stress response for vulnerable individuals. We evaluated the recovery index through the ReHo difference between two continuous time frames in the post‐task resting‐state runs, and a higher recovery ability was characterized by a greater difference between the two post‐stress time‐windows given the ReHo in the second segment recovers to a pre‐task baseline. We constrained the calculation within two‐segmented time‐windows to maintain the statistical power for each window; more sliding windows would be examined in the future.

Increased FC of the thalamus with the DMN and the sensory motor areas and decreased FC with the ECN indicate that, under acute stress, while the information related to self and essential sensory is up‐regulated, executive control signals may exert greater effort in controlling sensory input, and as a result, ECN might down‐regulate thalamus activity. The altered FC changes of the thalamus to the cortical regions especially the dlPFC imply the involvement of the thalamo‐cortical circuit during stress adaption, which is in align with findings in depression (Jiang et al., 2019; Kang et al., 2018). Further causality analyses might elucidate the FC changes among different networks. We did not observe activity or FC changes of the amygdala after acute stress; one potential explanation is that the activation of amygdala is more easily detected during the stressful tasks instead of resting‐state (Yin et al., 2011). In addition, not all studies found amygdala changes in subjects with depression (Li, Xu, & Lu, 2018; Wang, Yu, Wu, Wu, & Wang, 2019; Yao, Wang, Lu, Liu, & Teng, 2009).

Although our conclusions were drawn from individuals who were not clinically diagnosed as major depression, the ReHo difference and the opposite correlations of the ReHo/recovery index change and BDI scores between the higher and lower BDI group highly suggest the higher BDI group has distinct dynamic neural response patterns, which might be related to depression vulnerability. Thus, our study supports the feasibility of depression vulnerability detection by dynamic tracking of stress‐induced brain response and recovery.

Among stress‐related studies, structural and functional changes in the amygdala, mPFC and DMN (including hippocampus) have been most frequently reported and discussed (Dimitrov et al., 2018; Lau, Bigio, Zelli, McEwen, & Nasca, 2017; van Marle, Hermans, Qin, & Fernandez, 2009; van Marle et al., 2010; McEwen, Nasca, & Gray, 2016; Woon & Hedges, 2008). Few studies have focused on the thalamus. An fMRI study has demonstrated that acute stress elicits increased activation in the thalamus (Sinha et al., 2016) and increased FC between the hypothalamus (a key node in hypothalamic‐pituitary axis) and the thalamus (Magalhaes et al., 2018). Increased thalamus‐PCC FC has also been found during stress and returns to baseline later after stress (Vaisvaser et al., 2013). Animal studies have shown increased FC between the thalamus and the ventral tegmental area in stress‐susceptible animals (Magalhaes et al., 2018). While the majority of these studies found increased thalamus activity and thalamus‐DMN/midbrain FC under stress, a morphometric study of rats brain reported bilateral thalamus atrophy after severe stress exposure for 50 days but not in the amygdala (Yoshii et al., 2017), suggesting a long‐lasting detrimental effect of stress in the thalamus. These findings, together with our study, support the critical role of the thalamus in stress response although some are not entirely consistent with our results. However, the specific role of thalamus involved in stress reaction/recovery has not been fully understood; we believe that the activity/connectivity of the thalamus may reflect one's attention resources allocation between stress state and the homeostasis given it is a vital relay center between the amygdala and the mPFC and gateway for information filtering.

The current study has several potential limitations. First, the thalamic activity could be largely affected by individuals' state of alertness; without measurement of the consciousness level during the experiment, we cannot exclude this possibility with the current data. Second, stress‐induced changes did not fully recover to pre‐stress baseline at the end of the post‐stress resting run. Based on the study in the literature indicating that it might take 2 hr to recover from stress (Vaisvaser et al., 2013), further fMRI scan 2 hr after stress might help in better measuring the recovery ability. Third, the sample size of the lower and higher BDI groups is small. Our findings should be validated through a larger sample in future studies.

To summarize, by examining two widely recognized stressors, we found that acute stress‐induced changes in several networks including the limbic system, DMN, SN, and ECN. In particular, for the thalamus in the limbic system, we found that its regional connectivity (ReHo) could differentiate between higher and lower BDI groups, and the ReHo recovery index of the right thalamus could predict future depression severity. Therefore, the dynamic change in the thalamus regional connectivity might serve as a target for the prevention of depression. In addition, we proposed a recovery index for ReHo as a quantitative measure for brain recovery ability which could be extended to other brain activity metrics (ALFF, graph theory‐based measurements) as well for the vulnerability detection for psychiatric diseases.

## CONFLICT OF INTEREST

All authors declare no conflict of interest.

## Data Availability

The data that support the findings of this study are available from the corresponding author upon reasonable request.
