# Supplementary material for: Dynamic changes in thalamic connectivity following stress and its association with future depression severity
Source: Brain Behav. 2019 Oct 25;9(12):e01445. doi: 10.1002/brb3.1445 (PMC6908855; doi:10.1002/brb3.1445)
Supplement: Supplementary file 1 [file BRB3-9-e01445-s001.docx]

**Supplementary Materials**

**Experimental Design**

Each relaxing task run consisted of alternative presentation of 42s ‘+’ fixation block and 42s counting block (Fig. 1B) for 4 cycles. During the counting period, subjects were asked to count numbers from 1 to 10 along with breath.

The speech task also included 4 blocks cycles in each run, and each cycle included a 30s ‘+’ fixation block and a 32s speech block with 2s presentation of speech topic and 30s in-mind speech preparation (Fig. 1B). After each run and before the next run, one topic was randomly chosen for the subject to speak as they have prepared during the scanning. The experimenter was asked to give negative feedbacks to increase subject’s stress level.

During the math task (Fig. 1B), two 15s ‘+’ were shown at the beginning and at the end of each run as baseline, and forty trials (each trial lasted 5 seconds) of complex mathematic calculation problems were presented in between. Subjects were instructed to choose the correct answer from 4 choices by pressing a key in the keyboard. The math problems were mixed arithmetic expressions of addition, subtraction, multiplication, and division of no more than 4-digit integers (examples shown in Fig. 1B), and the 4 choices were made different only in the last 2 digits to reduce the difficulty level and ensure subjects’ task engagement. Yet, the tasks were difficult and the averaged task performance accuracy was 35% which was above the chance of 25% (out of four choices).

Subjects were trained before fMRI scans using shorter and easier tasks.

**Behavioral and Physiological Measures**

All statistical results are shown in the STable 1. In addition to the results of heart rate and cortisol levels in the higher BDI group which have been reported in the main text, there was a significant interaction of task by time in self-evaluated stress level (F_(10, 380)_ = 12.47, p < 0.001), and respiratory rate (F_(10, 380)_ = 9.45, p < 0.001). No significant effects were found for cortisol levels for all subjects (F_(6, 228)_ = 2.99, p = 0.24) (SFig. 1C). No significant effects were observed for state anxiety measurements (F_(2,76)_ = 0.82, p = 0.44). Paired t-test showed increased stress level after math task (T_(38)_ = 2.89, p = 0.003) but not speech (T_(38)_ = 0.59, p = 0.28) or relaxing task (T_(38)_ = -0.51, p = 0.31) (SFig. 1A). Respiratory rate did not change after stressful or relaxing tasks (SFig. 1B). No significant cortisol change was found for the lower BDI group (SFig. 1D).

**Results Summary for the Left Thalamus**

Consistent with the findings in the right thalamus, we found decreased ReHo in the left thalamus after both stressful tasks. The ReHo decrease was significantly greater in the higher BDI group and was associated with higher BDI scores in the higher BDI group. In addition, the FC of the left thalamus and the default mode network was increased after the stressful tasks, while the FC with the executive control network was decreased after the stressful tasks. The only difference from the findings in the right thalamus was that the ReHo recovery index in the left thalamus was not correlated with the BDI scores collected one-year-later.

**ReHo Changes in Left Thalamus post the Two Stressful Tasks and the Relationships with Risk of Depression**

ANOVA analysis revealed a significant interaction of time and task in the ReHo of the left thalamus (F_(2, 76)_ = 9.60, p < 0.001). SFig. 3A showed the ReHo in left thalamus from pre-task resting-state run, across 4 task runs to post-task resting-state run, decreased ReHo was also observed during both stressful tasks, and the ReHo in post-task runs was reduced (speech: T_(38)_ = 2.89, p = 0.002, math: T_(38)_ = 3.35, p = 0.01).

ReHo decrement, which was calculated as the sum of ReHo change during- and post-task relative to pre-task, was found significantly greater in the higher BDI group than that in the lower BDI group in both speech (T_(37)_ = 1.72, p = 0.05) and math (T_(37)_ = 1.74, p = 0.05) tasks (SFig. 3B). We found a negative correlation between the ReHo change in left thalamus and BDI score in the higher BDI group (speech: r = -0.62, p = 0.02; math: r = -0.62, p = 0.02), while the correlation was positive in the lower BDI group (speech: r = 0.55, p = 0.004; math: r = 0.46, p = 0.02) (SFig. 3C).

**Recovery Ability of Left Thalamus Activity Post Stress**

Regarding to the ReHo comparison between two time segments of post-stress resting run, there was a significant interaction effect of task and BDI groups (F_(2, 222)_ = 3.72, p = 0.03), however, there was no significant main or interaction effect of time. And paired t-test did not show any significant change among the SR and FR in either group (SFig. 3D). To be noted, the recovery index (RI) was found not correlated with current BDI or BDI scores at one-year follow-up (SFig. 3E) for any task conditions, which is different from the RI of the right thalamus.

**Common Changes in the Left Thalamus Connectivity Post Stress**

We found increased FC of the left thalamus with mPFC, PCC, bilateral superior temporal gyrus (STG), bilateral hippocampus and para-hippocampus, and sensorimotor areas (SFig. 4A), and decreased functional connectivity with right dlPFC, bilateral intra-parietal lobe (IPL) and thalamus itself (SFig. 4B). The connectivity change was not significantly different between the higher and lower BDI groups.

**Gender Effects on Stress-related Brain Changes**

Considering that the gender difference regarding to the stress response has been implicated in the literatures, a 3 tasks × 2 time-points × 2 BDI levels × 2 Genders 4-way mixed ANOVA was conducted to reveal the potential effects of gender on the stress-induced ReHo changes in bilateral thalamus, while there was a strong interaction between time and task as we reported in the manuscript, there is no significant main effect or interaction effect of gender (main effect of gender: left thalamus: F_(1, 210)_ = 1.69, p = 0.20; right thalamus: F_(1, 210)_ = 3.33, p = 0.07).

**STable 1 Statistical results for behavioral and physiological measures.**

|  | **Task × Time interaction** | **Post- vs. pre-relaxation, p value** | **Post- vs. pre-speech, p value** | **Post- vs. pre-math, p value** |
| --- | --- | --- | --- | --- |
| **Stress level** | F(10, 380) = 12.47  p < 0.001 | 0.31 | 0.28 | 0.003 |
| **Heart rate** | F(10, 380) = 13.56  p < 0.001 | 0.10 | 0.0004 | 0.0003 |
| **Respiratory rate** | F(10, 380) = 9.45  p < 0.001 | 0.51 | 0.19 | 0.81 |
| **Cortisol level** | Higher BDI  F (6, 78)= 2.28  p = 0.04 | Higher BDI  0.43 | Higher BDI  0.003 | Higher BDI  0.03 |
| **STAI-state** | F(2, 76) = 0.82  p = 0.44 | 0.96 | 0.95 | 0.13 |

STAI-state: Spielberger State Anxiety Inventory. Task × Time interaction for each measurement was evaluated through repeated ANOVAs; Post- versus pre-task difference was examined through paired t-test.


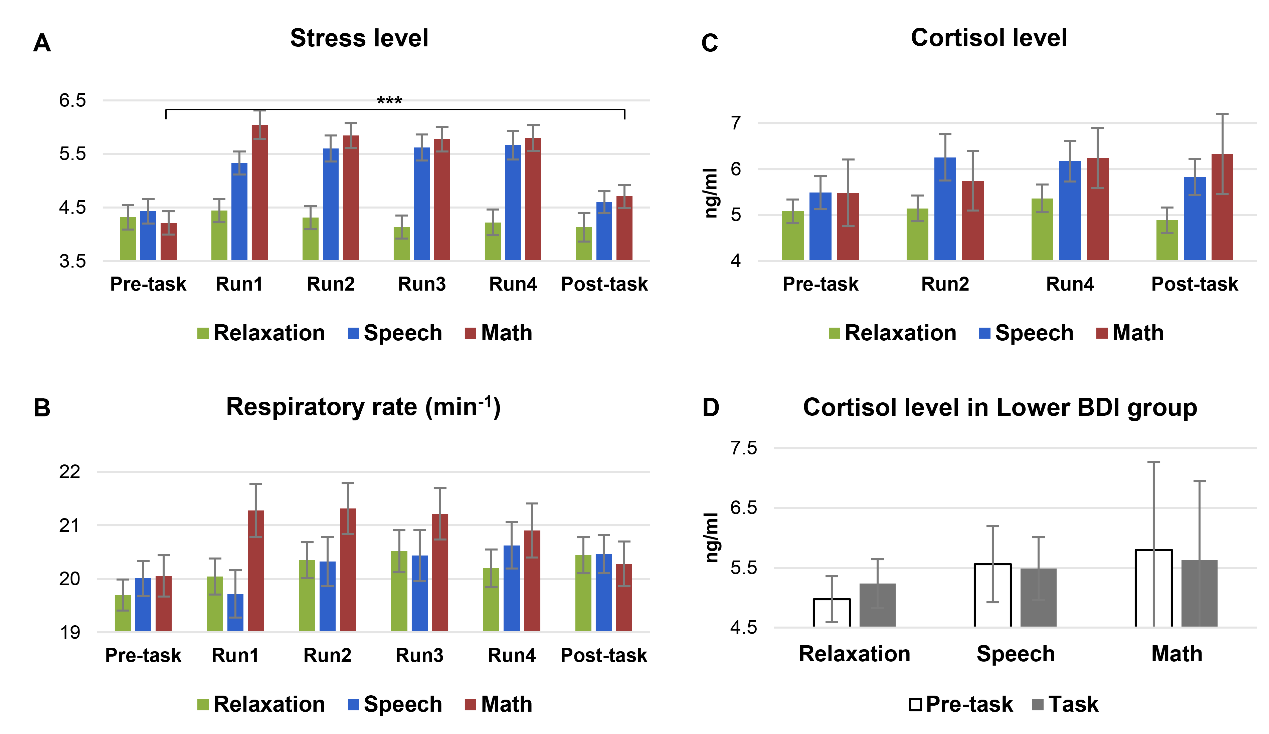


**SFig. 1. Stress induced (A) self-assessed stress level, (B) respiratory rate and (C) cortisol level changes over time from pre-task resting run, 4 task runs and post-task resting run under the two stressful tasks and the relaxation task. (D) Cortisol responses (mean change across and post-task vs. pre-task baseline) under the two stressful tasks and the relaxation task in the lower BDI group. ***: p < 0.001.**


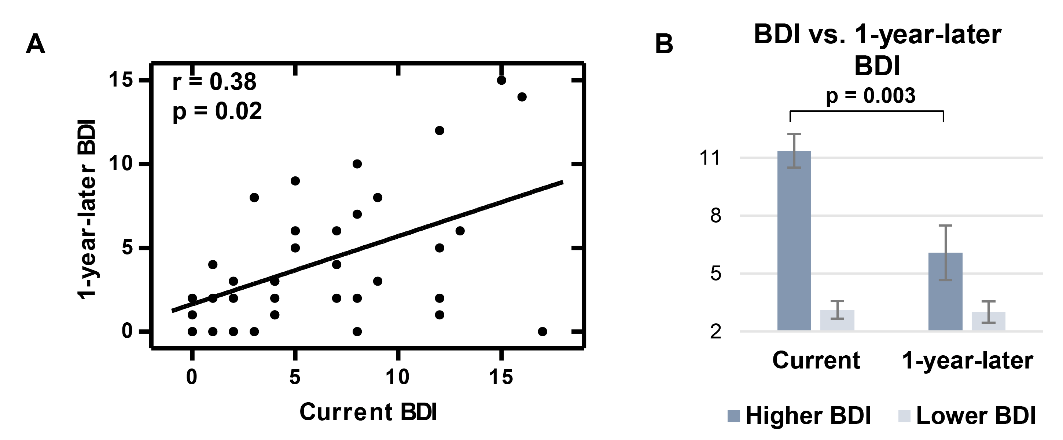


**SFig. 2. (A) Correlation between the current BDI and BDI score at 1-year-later follow-up; (B) The BDI score of individuals in the higher BDI group decreased 1 year after scanning.**

**
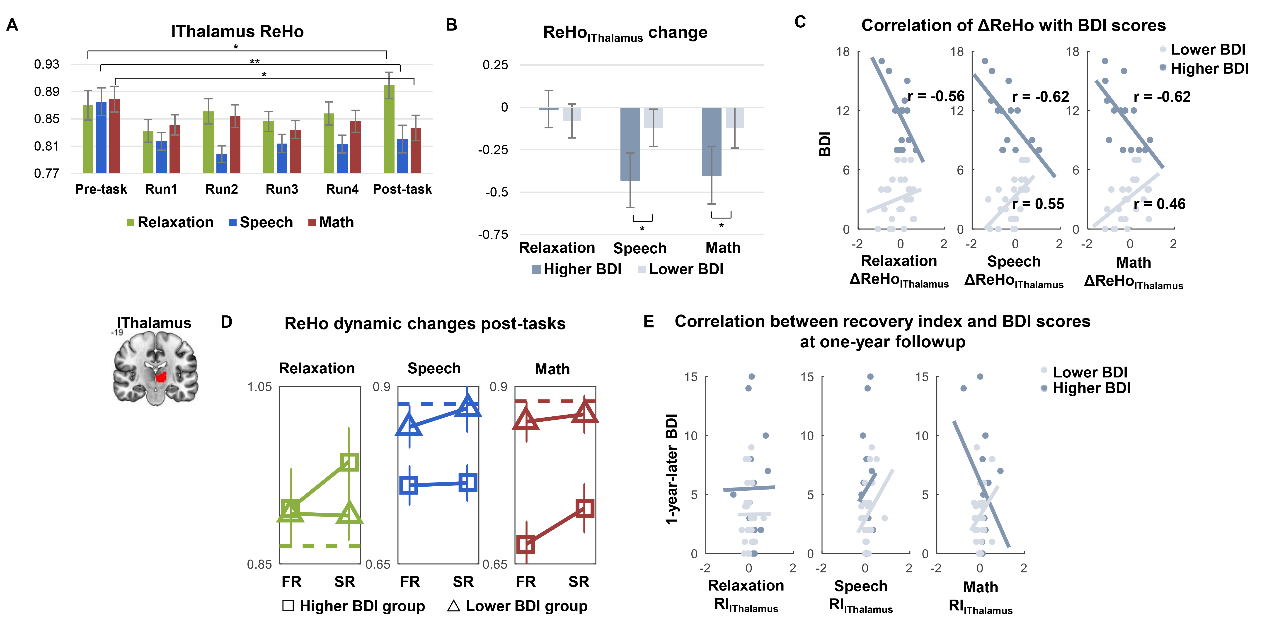
**

**SFig. 3. (A) Left thalamus (lThalamus) showed decreased ReHo during- and post- two stressful tasks but increased ReHo after relaxing task. (B) The ReHo decrease in the left thalamus during and after stressful tasks were significantly greater in the higher BDI group than that in the lower BDI group. (C) The ReHo change of the left thalamus was positively correlated with BDI in the lower BDI group but negatively correlated with BDI in the higher BDI group in both speech and math tasks. BDI score was negatively correlated with lThamulus ReHo change even post the relaxing task in the higher BDI group. (D) Comparison between the higher BDI group and lower BDI group in post-task lThalamus ReHo in the first half run (FR) and the second half run (SR), dotted lines indicate the pre-task ReHo in lThalamus. (E) The ReHo recovery index (RI) in the lThalamus post the two stressful tasks was not correlated with BDI scores at one-year follow-up in either higher BDI group or lower BDI group. *: p < 0.05, **: p < 0.01.**

**
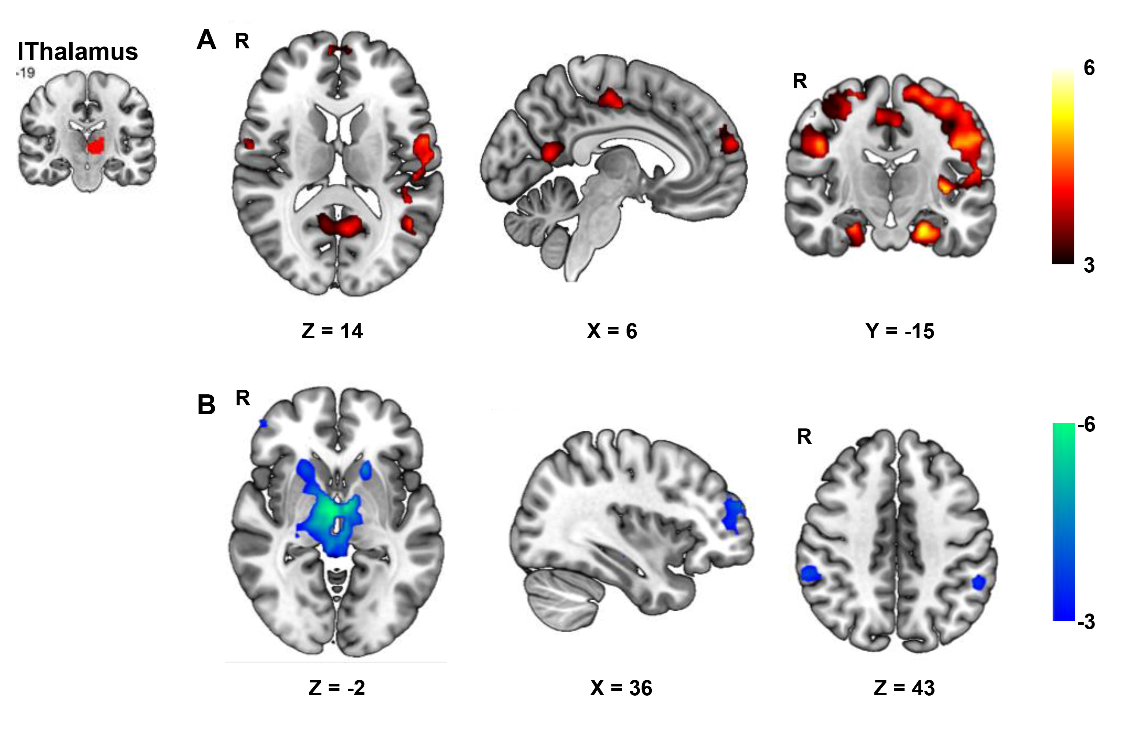
**

**SFig. 4. Stress induced functional connectivity changes of the left thalamus (lThalamus). (A) Increased functional connectivity of the lThalamus with mPFC (medial prefrontal cortex), mid-CC (middle cingulate cortex), PCC (posterior cingulate cortex), bilateral superior temporal gyrus, bilateral hippocampal & para-hippocampus, sensory-motor areas, and (B) decreased functional connectivity of the lThalamus with rdlPFC (right dorsal prefrontal cortex), bIPL (bilateral intraparietal lobe), putamen and thalamus itself.**
